# Thymoquinone Protects against Hyperlipidemia-Induced Cardiac Damage in Low-Density Lipoprotein Receptor-Deficient (LDL-R^−/−^) Mice via Its Anti-inflammatory and Antipyroptotic Effects

**DOI:** 10.1155/2020/4878704

**Published:** 2020-10-29

**Authors:** Zuo-Wei Pei, Ying Guo, Huo-Lan Zhu, Min Dong, Qian Zhang, Fang Wang

**Affiliations:** ^1^Department of Cardiology, Beijing Hospital, National Center of Gerontology, Institute of Geriatric Medicine, Chinese Academy of Medical Sciences, Beijing 100730, China; ^2^Peking Union Medical College, Chinese Academy of Medical Sciences, Beijing 100730, China

## Abstract

Hyperlipidemia is a risk factor for cardiac damage and cardiovascular disease. Increasing evidence has shown that dyslipidemia-related cardiac damage is associated with lipid accumulation, oxidative stress, and inflammation. Thymoquinone (TQ) is the major constituent of *Nigella sativa*, commonly known as black seed or black cumin, and is globally used in folk (herbal) medicine for treating and preventing a number of diseases and conditions. Several studies have shown that TQ can protect against cardiac damage. This study is aimed at investigating the possible protective effects of TQ on hyperlipidemia-induced cardiac damage in low-density lipoprotein receptor-deficient (LDL-R^−/−^) mice. Eight-week-old male LDL-R^−/−^ mice were randomly divided into normal diet (ND), high-fat diet (HFD), and HFD and TQ (HFD+TQ) groups and were fed the different diets for eight weeks. Blood samples were obtained from the inferior vena cava in serum tubes and stored at -80°C until use. Some cardiac tissues were fixed in 10% formalin and then embedded in paraffin for histological evaluation. The remainder of the cardiac tissues was snap-frozen in liquid nitrogen for mRNA preparation or immunoblotting. The levels of metabolism-related factors, such as total cholesterol (TC), low-density lipoprotein-cholesterol (LDL-c), and high-sensitivity C-reactive protein (hs-CRP), were decreased in the HFD+TQ group compared with those in the HFD group. Periodic acid-Schiff staining demonstrated that lipid deposition was lower in the HFD+TQ group than in the HFD group. The expression of pyroptosis indicators (NOD-like receptor 3 (NLRP3), interleukin- (IL-) 1*β*, IL-18, and caspase-1), proinflammatory factors (IL-6 and tumor necrosis factor alpha (TNF-*α*)), and macrophage markers (cluster of differentiation (CD) 68) was significantly downregulated in the HFD+TQ group compared with that in the HFD group. Our results indicate that TQ may serve as a potential therapeutic agent for hyperlipidemia-induced cardiac damage.

## 1. Introduction

Cardiovascular disease (CVD) is a major cause of mortality and morbidity worldwide [1, 2]. Among the causes of cardiovascular disease, hyperlipidemia is a critical damage-inducing factor [3]; individuals with hyperlipidemia have a higher risk for CVD than those with normal cholesterol levels [4]. Hyperlipidemia is characterized by an increase in triglyceride (TG), total cholesterol (TC), and low-density lipoprotein-cholesterol (LDL-c) and/or a decrease in high-density lipoprotein-cholesterol (HDL-C) [5]. Increasing evidence has shown that this abnormality in lipid metabolism causes lipid accumulation, oxidative stress, and inflammation, leading to cardiac damage [6, 7]. Several researchers have investigated various drugs for the treatment of hyperlipidemia, such as statins; however, as these drugs are related to the development of cell resistance and are associated with adverse effects, new methods for treating hyperlipidemia are needed. Therefore, the use of natural hypolipidemic drugs may constitute a promising strategy for the prevention and treatment of hyperlipidemia [8].

Thymoquinone (TQ) is the major constituent of *Nigella sativa* [9], commonly known as black seed or black cumin, and is globally used in folk (herbal) medicine for treating and preventing a number of diseases and conditions [10]. Previous studies have reported that TQ suppresses chronic cardiac inflammation [11] and regulates the expression of factors, such as vascular endothelial growth factor and nuclear factor-erythroid-2-related factor 2, thereby improving the antioxidant potential of the cardiac muscle. In addition, TQ alleviates diabetes-associated oxidative stress in cardiac tissues [12]. Furthermore, several studies have shown that the protective effect of TQ against cardiac damage, such as in cases of ischemic damage [13] and acute abdominal aortic ischemia-reperfusion injury [14], is mediated via the pyroptosis pathway [15]. Recently, pyroptosis, an inflammatory form of programmed cell death [16], has been gaining increasing attention, especially in relation to hyperlipidemia [17, 18]; however, the pathophysiological mechanisms underlying the relationship between hyperlipidemia and cardiac damage are not yet fully understood.

Therefore, in this study, we investigated the role of TQ in hyperlipidemia-induced cardiac damage in a low-density lipoprotein receptor-deficient (LDL-R^−/−^) mouse model and the possible underlying mechanisms.

## 2. Material and Methods

### 2.1. Animal Model

LDL-R^−/−^ mice were purchased from Beijing Vital River Lab Animal Technology Co., Ltd. (Beijing, China; No. 2019001103468). All mice were bred in a room with a 12/12 h light-dark cycle at a controlled temperature (24–26°C). Male LDL-R^−/−^ mice (8-week-old) were randomly divided into the following three groups: the normal diet (ND, *n* = 8), high-fat diet (HFD, *n* = 8), and high-cholesterol diet+50 mg/kg/day of TQ (HFD+TQ, *n* = 8) groups [19]. Thymoquinone was dissolved in corn oil solution [20]. The HFD contained 1.2% cholesterol and 21% fat. The experimental diet was purchased from Shanghai Slac Laboratory Animal Co., Ltd. (Shanghai, China). Mice in all groups were fed the appropriate diet for eight weeks. Blood samples were acquired from the inferior vena cava in serum tubes and stored at -80°C until use. Cardiac tissues were fixed in 10% formalin and embedded in paraffin for histological evaluation. The remaining cardiac tissues were snap-frozen in liquid nitrogen for mRNA isolation and immunoblotting analyses. All experimental procedures were approved by the National Laboratory Animal Management Regulations and the Beijing Hospital Laboratory Animal Management Regulations.

### 2.2. Biochemical Measurements

Sera were separated from the collected blood samples using centrifugation at 3000 rpm for 15 min. The levels of TC, low-density lipoprotein-cholesterol (LDL-c), and high-sensitivity C-reactive protein (hs-CRP) in the serum were detected using the total cholesterol, low-density lipoprotein-cholesterol, and high-sensitivity C-reactive protein assay kit, according to the manufacturer's instructions. The serum IL-6 and TNF-*α* levels were determined using an ELISA kit according to the manufacturer's instructions.

### 2.3. Hematoxylin and Eosin Staining

The cardiac tissues were fixed with 10% buffered formalin for 30 min and then dehydrated in 75% ethanol overnight, followed by paraffin embedding. Serial sections (4 *μ*m) were stained with hematoxylin and eosin for pathological analysis.

### 2.4. Periodic Acid-Schiff (PAS) Staining

Cardiac tissues from each group were stored in 10% formalin, dehydrated in an ascending alcohol series (75, 85, 90, and 100% alcohol, 5 min each), and then embedded in paraffin wax. Paraffin sections (4 *μ*m thick), sliced from these paraffin-embedded tissue blocks, were then deparaffinized via immersion in xylene (three times, 5 min each) and rehydrated using a descending alcohol series (100, 90, 85, and 75% alcohol, 5 min each). Samples were stained with PAS stain to investigate the changes in cardiac morphology. Red staining indicates lipid deposition.

### 2.5. Immunohistochemistry

Immunohistochemistry was performed according to the manufacturer's instructions using antibodies against cluster of differentiation (CD) 68 and CD36. The results were visualized using an Olympus microscope (Olympus, Tokyo, Japan). The NIH ImageJ software was used for positive cell quantification. Furthermore, statistical analysis of the differences between samples was performed.

### 2.6. Western Blotting

The protein samples obtained from cardiac tissues were separated using 10% sodium dodecyl sulfate-polyacrylamide gel electrophoresis (SDS-PAGE) and then transferred to polyvinylidene fluoride membranes. The membranes were blocked in Tris-buffered saline with 0.1% Tween-20 (TBS-T) containing 5% skimmed milk and then incubated overnight with gentle shaking at 4°C in a diluent containing primary antibodies against NLRP3, IL-18, IL-1*β*, caspase-1, P-ERK, NF-*κ*B, p-38, and anti-*β*-actin. The membranes were then incubated with a secondary antibody for 1 h. This analysis was performed independently three times. Protein levels were expressed as protein/*β*-actin ratios to minimize the loading differences. The relative signal intensity was quantified using the NIH ImageJ software.

### 2.7. RNA Isolation and Real-Time PCR (qPCR)

Total RNA was isolated from cardiac tissues, and complementary DNA (cDNA) was synthesized using the TransScript One-Step gDNA Removal and cDNA Synthesis SuperMix kit according to the manufacturer's protocol. Gene expression was quantitatively analyzed using qPCR and the TransStart Top Green qPCR SuperMix kit. *β*-Actin was amplified and quantitated in each reaction to normalize the relative amounts of the target genes. Primer sequences are listed in Table 1.

### 2.8. Statistical Analysis

The normality of the data was tested. All data are presented as the mean ± standard error of mean (SEM). Statistical analysis was performed using the SPSS software version 23.0 (SPSS Inc., Chicago, IL, USA). Intergroup variation was measured using one-way analysis of variance followed by Tukey's test. The threshold for statistical significance was set at *P* < 0.05.

### 2.9. Materials and Reagents

The materials and reagents were the following: total cholesterol, low-density lipoprotein-cholesterol, and high-sensitivity C-reactive protein assay kit (Nanjing Jiancheng Bioengineering Institute, Nanjing, China); IL-6 and TNF-*α* ELISA kit (Proteintech, Wuhan, China); immunohistochemistry staining kit (Zsbio, Beijing, China); primary antibodies against CD68 (rabbit anti-CD68 antibody, 1 : 200; Proteintech, Wuhan, China); CD36 (rabbit anti-CD36 antibody, 1 : 200; Proteintech); polyvinylidene fluoride membranes (Immobilon, Millipore, Billerica, MA, USA); diluent (P0023A; Beyotime); NOD-like receptor 3 (NLRP3; rabbit anti-NLRP3 antibody, 1 : 1000; Boster, Wuhan, China); interleukin- (IL-) 18 (rabbit anti-IL-18 antibody, 1 : 1000; Proteintech); IL-1*β* (rabbit anti-IL-1*β* antibody, 1 : 1000; Arigo, Hamburg, Germany); caspase-1 (rabbit anti-caspase-1 antibody, 1 : 1000; Proteintech); phosphoextracellular signal-related kinase (P-ERK; rabbit anti-P-ERK, 1 : 1000; Proteintech); NF-*κ*B (NF-*κ*B; rabbit anti-NF-*κ*B, 1 : 1000; Proteintech); p-38 (p-38; rabbit anti-p-38, 1 : 1000; Proteintech); anti-*β*-actin (1 : 1000; Proteintech); secondary antibody (anti-rabbit Ig-G, 1 : 1000; Cell Signaling Technology); cDNA Synthesis SuperMix kit (Transgen, Beijing, China); qPCR; and the TransStart Top Green qPCR SuperMix kit (Transgen).

## 3. Results

### 3.1. Metabolic Characterization

At the end of the experiment, all mice survived. The metabolic characteristics of LDL-R^−/−^ mice after eight weeks of different treatments are summarized in Table 2. The heart/body weight ratio did not change in the three groups. The TC, LDL-c, hs-CRP, IL-6, and TNF-*α* levels were markedly increased in the HFD group but significantly decreased in the HFD+TQ group.

### 3.2. TQ Reduced the HFD-Induced Cardiac Damage

**Figure 1:**
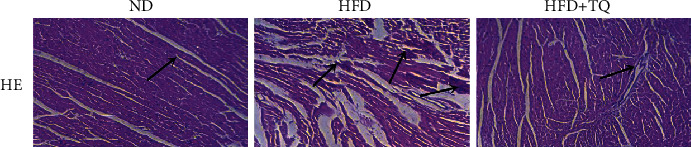
Effect of TQ on hyperlipidemia-induced histopathological changes in the cardiac tissues. HE staining in cardiac tissues of three groups with different treatments. Magnification 40x. The arrows indicate damage. *n* = 3 per group.

**Figure 2:**
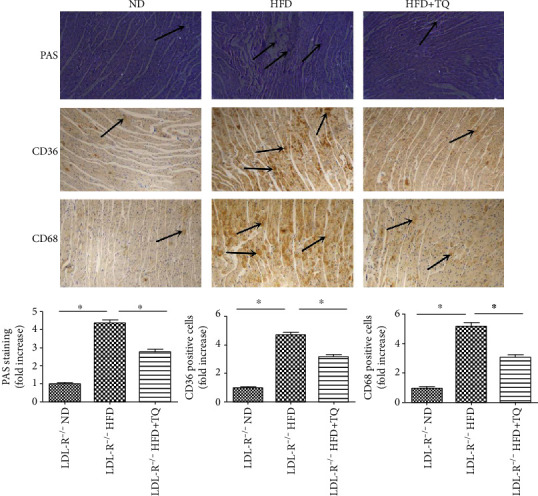
Effect of TQ on hyperlipidemia-induced lipid accumulation in the cardiac tissues. PAS, CD36, and CD68 staining in cardiac tissues of three groups with different treatments. Magnification 40x. The arrows indicate damage. *n* = 3 per group. Bar graph showing PAS-, CD36-, and CD68-positive cells. ^∗^*P* < 0.05.

To evaluate inflammatory cell infiltration into cardiac tissue, hematoxylin and eosin staining was performed (Figure 1). The HFD+TQ group showed markedly reduced inflammatory cell infiltration into cardiac tissue compared with the HFD group, indicating that TQ reduced HFD-induced cardiac damage. To evaluate lipid accumulation in cardiac tissue, we evaluated the PAS staining and the expression of CD36 and CD68 (Figure 2). Increased lipid retention was detected in the cardiac tissues of HFD-fed mice. Interestingly, the HFD+TQ group showed markedly reduced lipid deposition in the cardiac tissue compared with the HFD group.

### 3.3. TQ Reduced the HFD-Induced Expression of Proinflammatory Cytokines in Mouse Cardiac Tissues

**Figure 3:**
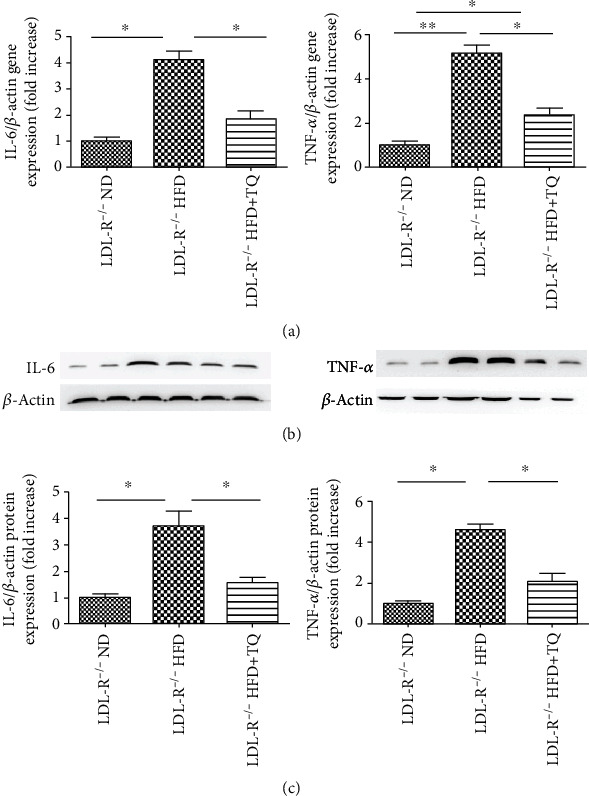
Proinflammatory gene and protein expression in the cardiac tissue. (a) Relative mRNA expression of IL-6 and TNF-*α* in cardiac tissue of three groups with different treatments. (b) Immunoblotting for IL-6 and TNF-*α* protein expression in cardiac tissues. (c) Bar graph showing quantification of IL-6 and TNF-*α* protein expression. Data are given as the means ± SEM; *n* = 5-6 in each group. ^∗^*P* < 0.05; ^∗∗^*P* < 0.01.

To examine the involvement of proinflammatory cytokines in the cardiac tissues of the three mouse groups, the mRNA and protein expression levels of IL-6 and tumor necrosis factor alpha (TNF-*α*) were measured using qPCR and western blotting (Figure 3). Although IL-6 and TNF-*α* mRNA and protein were upregulated in the HFD group, upregulation was attenuated in the HFD+TQ group.

### 3.4. TQ Reduced the HFD-Induced Pyroptosis in Mouse Cardiac Tissues

**Figure 4:**
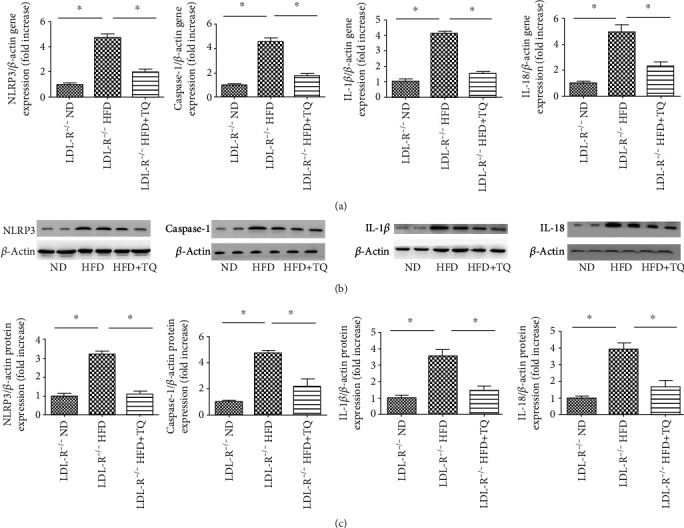
Pyroptosis expression in the cardiac tissues. (a) Relative mRNA expression of NLRP3, caspase-1, IL-1*β*, and IL-18 in cardiac tissue of three groups with different treatments. (b) Immunoblotting for NLRP3, caspase-1, IL-1*β*, and IL-18 protein expression in cardiac tissues. (c) Bar graph showing quantification of NLRP3, caspase-1, IL-1*β*, and IL-18 protein expression. Data are given as the means ± SEM; *n* = 5-6 in each group. ^∗^*P* < 0.05.

To evaluate pyroptosis in cardiac tissues, we examined the mRNA and protein expression of the pyroptosis indicators NLRP3, caspase-1, IL-1*β*, and IL-18 (Figure 4). The mRNA levels of NLRP3, caspase-1, IL-1*β*, and IL-18 were significantly downregulated in the HFD+TQ group compared with those in the HFD group (Figure 4(a)). Western blotting (Figure 4(b)) demonstrated that the protein levels of NLRP3, caspase-1, IL-1*β*, and IL-18 were markedly reduced in cardiac tissues of the HFD+TQ group compared with those in the HFD group (Figures 4(b) and 4(c)). These results indicate that TQ reduced the HFD-induced upregulation of NLRP3, caspase-1, IL-1*β*, and IL-18 expression.

### 3.5. TQ Reduced the HFD-Induced Increase in P-ERK, NF-*κ*B, and p-38 Levels in Mouse Cardiac Tissues

**Figure 5:**
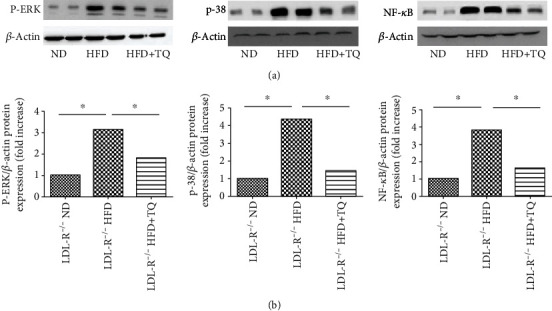
Phospho-ERK, NF-*κ*B, and p-38 expression in the cardiac tissues. (a) Immunoblotting for phospho-ERK, NF-*κ*B, and p-38 levels in cardiac tissues. (b) Bar graph shows the quantification of phospho-ERK, NF-*κ*B, and p-38 levels. Data are given as the means ± SEM; *n* = 3 in each group. ^∗^*P* < 0.05.

To investigate the effect of TQ on the regulation of the ERK, NF-*κ*B, and p-38 signaling pathways, we analyzed the P-ERK, NF-*κ*B, and p-38 levels in the respective treatment groups using western blotting (Figure 5). P-ERK, NF-*κ*B, and p-38 levels were higher in the HFD group than in the ND group, and the HFD+TQ group exhibited significantly lower P-ERK, NF-*κ*B, and p-38 levels than the HFD group.

## 4. Discussion

**Figure 6:**
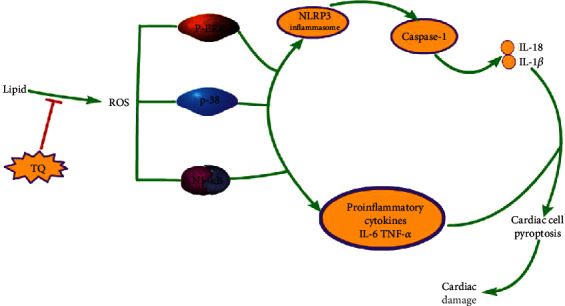
The effect of TQ on the cardiac damage caused by hyperlipidemia.

The present study demonstrates that TQ has a protective effect on hyperlipidemia-induced progressive lipid deposition, proinflammatory cytokine expression, and pyroptosis. The specific mechanism underlying this effect is shown in Figure 6.

The results of metabolic characterization indicated that the TC and LDL-c levels were increased in the HFD group compared to those in the ND group. These results are consistent with those obtained by Kolbus et al. [21]. Interestingly, the TC and LDL-c levels in the HFD+TQ group were significantly lower than those in the HFD group. Several clinical studies have indicated that hs-CRP can serve as a biomarker in cardiovascular event risk prediction [22, 23]. Our results show that the HFD+TQ group had markedly reduced serum hs-CRP levels than those in the HFD group, indicating that TQ influences cholesterol metabolism and hs-CRP levels.

Hyperlipidemia promotes macrophage accumulation and lipid deposition in cardiac tissues [24]. Cellular lipid homeostasis involves the regulation of the influx, synthesis, catabolism, and efflux of lipids. An imbalance in these processes can result in the conversion of macrophages into foam cells [25]. The CD68 marker identifies a population of macrophages; CD68-positive cells are often observed in infiltrating cardiac tissues [25]. In addition, studies have shown that oxLDL can stimulate the expression of scavenger receptors (CD36, low-density lipoprotein receptor 1, and scavenger receptor A) in monocyte-derived macrophages, thereby inducing macrophages to form foam cells [26, 27]. Huang and his colleagues demonstrated that inhibiting the expression of scavenger receptors, such as CD36, inhibits the formation of foam cells [28]. The results of our lipid deposition assays showed that CD68 and CD36 expression and PAS staining were significantly increased in the LDL-R^−/−^ HFD group compared with those in the LDL-R^−/−^ ND group; however, this damage was significantly inhibited in the HFD+TQ group, indicating that TQ inhibits the cardiac damage caused by hyperlipidemia by inhibiting lipid deposition and the conversion of macrophages to foam cells.

High expression of proinflammatory cytokines, known to contribute to cardiac damage, has been reported in hyperlipidemia [29, 30]. Our study showed that the expression of IL-6 and TNF-*α* was reduced in the HFD+TQ group compared with that in the HFD group, indicating that TQ downregulated the HFD-induced expression of IL-6 and TNF-*α*. As early as 1997, Mutabagani and El-Mahdy confirmed the anti-inflammatory activity of TQ in rats [31]. This study showed that hyperlipidemia can cause cardiac damage by increasing the expression of proinflammatory cytokines and that TQ can inhibit this damage through its anti-inflammatory effects.

Pyroptosis is a novel programmed cell death mechanism. Recent studies have reported that pyroptosis contributes to the development of hyperlipidemia. Pyroptosis induction is closely associated with the activation of the NLRP3 inflammasome, which has been linked to key cardiovascular risk factors, including hyperlipidemia [32, 33]. A significant decrease in atherosclerotic lesion size was also observed in the aortic sinus of HFD-fed LDL-R^−/−^ mice reconstituted with NLRP3 knockout bone marrow cells [32]. In addition, previous studies have shown that NLRP3 recruits caspase-1, leading to the activation of caspase-1, maturation and secretion of IL-1*β* and IL-18, and initiation of pyroptosis [34–37]. Our results showed that the cardiac tissues in the HFD+TQ group expressed markedly reduced levels of NLRP3, caspase-1, IL-1*β*, and IL-18 compared with those in the HFD group, indicating that TQ downregulated HFD-induced pyroptosis.

Oxidative stress and inflammation are important causes of cardiovascular disease [38, 39]. Studies have shown that hyperlipidemia causes cardiac damage by increasing oxidative stress and that reactive oxygen species (ROS) play an important role in this damage [40]. Furthermore, it has been confirmed that excessive accumulation of ROS can transmit the signal to downstream ROS-sensitive signaling pathways, such as NF-*κ*B, ERK1/2, p38 MAPK, and autophagy-related signaling to induce pathological cardiac hypertrophy [41–44]. Therefore, in our study, we analyzed the protein levels of NF-*κ*B, p-38, and P-ERK in the respective treatment groups using western blotting. The results showed that the NF-*κ*B, p-38, and P-ERK levels were higher in the HFD group than in the ND group and that the HFD+TQ group exhibited significantly lower NF-*κ*B, p-38, and P-ERK levels than the HFD group. These results indicate that hyperlipidemia causes cardiac damage via ROS-sensitive signaling pathways (NF-*κ*B, p-38, and P-ERK) and that TQ can reduce the cardiac damage caused by hyperlipidemia by inhibiting these pathways. Zhang et al. confirmed that hydrogen (H_2_) inhibits isoproterenol-induced cardiac hypertrophy via the NF-*κ*B, p-38, and P-ERK pathways [45]. In addition, Xu et al. showed that TQ reduces cardiac damage via the phospho-ERK pathway [19], whereas Tabeshpour et al. showed that TQ inhibits the expression of the p-38 pathway [46].

## 5. Conclusions

The reduced lipid deposition and pyroptosis and downregulated proinflammatory cytokine expression found in mice fed TQ in our study establish that TQ contributes to the mitigation of hyperlipidemia-induced cardiac damage. These findings provide new insights into the role of TQ in hyperlipidemia-induced cardiac damage and introduce the possibility of a novel therapeutic intervention for treating CVDs.

## Figures and Tables

**Table 1:** Primer oligonucleotide sequences.

Gene	Primers
TNF-*α* (ID: Y00467.1)	F: 5′-TCTCATGCACCACCATCAAGGACT-3′
	R: 5′-ACCACTCTCCCTTTGCAGAACTCA-3′
IL-6 (ID: XM_032905335.1)	F: 5′-TACCAGTTGCCTTCTTGGGACTGA-3′
	R: 5′-TAAGCCTCCGACTTGTGAAGTGGT-3′
NLRP3 (ID: XM_021213477.2)	F: 5′-CTGCGGACTGTCCCATCAAT-3′
	R: 5′-AGGTTGCAGAGCAGGTGCTT-3′
IL-1*β* (ID: NM_008361.4)	F: 5′-TGCCACCTTTTGACAGTGAT-3′
	R: 5′-TGTGCTGCTGCGAGATTTGA-3′
IL-18 (ID: AC140070.3)	F: 5′-ATGGCTGCTGAACCAGTAGAAG-3′
	R: 5′-CAGCCATACCTCTAGGCTGGC-3′
Caspase-1 (ID: XM_032898880.1)	F: 5′-AACCAGGAGAATGTTTCCAACCT-3′
	R: 5′-AAACACCAGGCCAAGCTTCTT-3′
*β*-Actin (ID: XM_032887061.1)	F: 5′-CGATGCCCTGAGGGTCTTT-3′
	R: 5′-TGGATGCCACAGGATTCCAT-3′

Abbreviations: TNF-*α*: tumor necrosis factor-*α*; IL-6: interleukin-6; NLRP3: nucleotide-binding and oligomerization domain-like receptor 3; IL-18: interleukin-18; IL-1*β*: interleukin-1*β*.

**Table 2:** Metabolic data from the four groups after 8 weeks of dietary treatment.

	LDL-R^−/−^ ND	LDL-R^−/−^ HFD	LDL-R^−/−^ HFD+TQ
Heart/BW (mg/g)	4.175 ± 0.1966	3.814 ± 0.3045	4.483 ± 0.08
TC (mmol/L)	8.5 ± 1.207^∗^	34.01 ± 2.318	14.08 ± 0.7108^∗^
LDL-c (mmol/L)	3.938 ± 0.1281^∗^	23.88 ± 1.651	6.783 ± 0.6817^∗^
hs-CRP (ng/dL)	58.5 ± 4.252^∗^	221.2 ± 13.43	111.7 ± 10.19^∗^^#^
IL-6 (pg/mL)	18.1 ± 1.9266^∗^	79.956 ± 4.0109	43.33 ± 6.9251^∗^^#^
TNF-*α* (pg/mL)	19.156 ± 4.7569^∗^	88.708 ± 3.6499	45.262 ± 4.0316^∗^^#^

Abbreviations: BW: body weight; TC: total cholesterol; LDL-c: low-density lipoprotein-cholesterol; IL-6: interleukin-6; TNF-*α*: tumor necrosis factor-*α*. Data are means ± SEM; *n* = 5-6 per group. ^∗^*P* < 0.05 vs. LDL-R^−/−^ HFD; ^#^*P* < 0.05 vs. LDL-R^−/−^ ND.

## Data Availability

Our dates are presented the article.

## Abbreviations

HFD: High-fat diet
hs-CRP: High-sensitivity C-reactive protein
IL: Interleukin
LDL-R^−/−^: Low-density lipoprotein receptor-deficient
LDL-c: Low-density lipoprotein-cholesterol
ND: Normal diet
NLRP3: NOD-like receptor 3
Nrf2: Nuclear factor-erythroid-2-related factor 2
P-ERK: Phosphoextracellular signal-related kinase
SDS-PAGE: Sodium dodecyl sulfate-polyacrylamide gel electrophoresis
SEM: Standard error of mean
TC: Cholesterol
TNF-*α*: Tumor necrosis factor alpha
TQ: Thymoquinone.

## Additional Points

The submission of the manuscript is in the preprint.
